# Low-Cost PVD Shadow Masks with Submillimeter Resolution from Laser-Cut Paper

**DOI:** 10.3390/mi11070676

**Published:** 2020-07-11

**Authors:** Farzad Elhami Nik, Isabelle Matthiesen, Anna Herland, Thomas E. Winkler

**Affiliations:** 1 Division of Micro- and Nanosystems, KTH Royal Institute of Technology, 11428 Stockholm, Sweden; farzad.elhami@mail.polimi.it (F.E.N.); imat@kth.se (I.M.); 2 Department of Electronics, Information and Bioengineering, Politecnico di Milano, 20133 Milan, Italy; 3 AIMES, Department of Neuroscience, Karolinska Institute, 17177 Solna, Sweden

**Keywords:** shadow mask, stencil lithography, CO_2_ laser, paper, metal deposition

## Abstract

We characterize an affordable method of producing stencils for submillimeter physical vapor deposition (PVD) by using paper and a benchtop laser cutter. Patterning electrodes or similar features on top of organic or biological substrates is generally not possible using standard photolithography. Shadow masks, traditionally made of silicon-based membranes, circumvent the need for aggressive solvents but suffer from high costs. Here, we evaluate shadow masks fabricated by CO_2_ laser processing from quantitative filter papers. Such papers are stiff and dimensionally stable, resilient in handling, and cut without melting or redeposition. Using two exemplary interdigitated electrode designs, we quantify the line resolution achievable with both high-quality and standard lenses, as well as the positional accuracy across multiple length scales. Additionally, we assess the gap between such laser-cut paper masks and a substrate, and quantify feature reproduction onto polycarbonate membranes. We find that ~100 µm line widths are achievable independent of lens type and that average positional accuracy is better than ±100 µm at 4”-wafer scale. Although this falls well short of the micron-size features achievable with typical shadow masks, resolution in the tenths to tens of millimeters is entirely sufficient for applications from contact pads to electrochemical cells, allowing new functionalities on fragile materials.

## 1. Introduction

Microelectromechanical systems (MEMS) are pervading more and more areas of our lives, from initially mainly as vehicular sensors to on-body physiological monitoring in recent years [1]. Yet, standard photolithographic processes remain limited in their material compatibility, creating the need for cheap and scalable fabrication on unconventional substrates, such as soft and flexible materials. With respect to additive processes to add, e.g., sensing functionalities to such materials, this has led to the implementation and adaptation of various printing processes, from inkjet printing to screen printing [2]. Although highly flexible, these processes are limited by their reliance on liquid ink and thus material quality, compared to the physical vapor deposition (PVD) of traditional MEMS.

PVD on unconventional substrates can be facilitated with the use of shadow mask lithography (SML), similar to the stencils used in screen printing [3,4]. In SML, desired geometries are initially produced in a negative shadow mask—analogous to a photomask, but with physical instead of merely optical occlusions. During the PVD process, this mask is placed in close contact with the substrate so that atoms or ions can pass through the mask apertures to reproduce the intended features. The most significant advantage of SML is that it does not require coating and patterning photoresist on target substrates, which consequently eliminates substrate exposure to potentially harmful solvents, heat, or UV radiation. This makes SML an ideal procedure to pattern on not only biological samples and materials like thin plastic films that are sensitive to solvents but also water-soluble sheets. Additionally, the simpler process flow reduces both cost and time required for fabrication.

Rigid shadow masks, generally made of Si/SiN-based membranes or metal films, are the primary type in use. The former are typically fabricated using traditional bulk and surface micromachining, and allow for mask features in line with the lithography method used (i.e., down to nanometer-scale with electron beam or deep UV exposure) [3,4]. Metal-based masks arise out of surface-mount technology (SMT), where these types of stencils are used to apply solder paste to printed circuit boards. They are typically fabricated using similar photolithography approaches to Si/SiN masks or by laser cutting [5]. SML has enabled micro- and even nanoscale PVD on fragile materials, from gold metamaterial antennas on silk and paper [6,7] to indium–tin-oxide (ITO)-based organic light-emitting diodes (OLEDs) on plastic films [8]. However, the fabrication of rigid shadow masks along with their mounting in a suitable frame (mainly needed for metal films) is exceedingly expensive and time consuming, requiring a wide range of process equipment as well as relevant training. Commercial costs for a 4” wafer-suitable mask can easily exceed €1000, even at relatively low resolution (5–10 µm). This presents a significant limitation, particularly in an academic setting where rapid design iteration is often needed.

Although high mask resolution is naturally critical to nanofabrication [4] and can also be beneficial for microfabrication [9], we note that many applications do not actually require the high resolution afforded by traditional shadow masks. Zhao et al. recently published an innovative wearable multianalyte sensor system, enabled by fabrication on top of an adhesive conductive film [10]. The SML PVD gold underlying all active sensor elements has a minimum feature size of ~0.5 mm. Traditional three-electrode electrochemical cells with >100 µm features are indeed a common example of SML, e.g., on permeable cell culture supports [11] or on paper [12]. Ishikawa et al. prepared contact pads (>100 µm) by SML on top of carbon nanotubes for algal detection [13]. Resistive sensors on collagen films (>1 mm) [14] and capacitive sensors in polydimethylsiloxane (PDMS; >150 µm) [15] were similarly realized by SML.

It is in light of these limitations and needs that we consider alternative materials and fabrication methods for shadow masks (Table 1). Photolithographic shadow mask fabrication, independent of the materials used, is not well-suited for low-cost academic prototyping. Direct photomask writers and robotic lithography systems could facilitate high turnaround, but remain resource and training intensive and uncommon in academic cleanrooms. Laser machining, on the other hand, deserves further consideration, since it is a single-step process (excepting the possible need for mounting in a frame, required to keep thin membranes under tension). Direct photoablation of a wide range of materials is possible with excimer or ultrafast lasers. This “cold” processing yields high-quality cuts (only limited by laser spot size) but equipment costs remain prohibitive [16]. Processing with other laser systems is largely thermal (i.e., at least some of the material will undergo a solid-to-liquid transition), including the neodymium-doped yttrium aluminum garnet (Nd:YAG) lasers often used for commercial steel or nickel stencil fabrication [17]. Notably, this also includes CO_2_ lasers, by far the most affordable type of laser processing available (used CO_2_ laser cutters can be found from ~€100; other laser types from ~€10,000).

In terms of material alternatives, nontraditional shadow masks have been introduced in recent years, e.g., from polydimethylsiloxane (PDMS) [18] or polyimide [19] (both examples using photolithography for mask fabrication). The driving motivation was often to obtain compliant (zero-gap) masks to alleviate the blurring effect arising from the small (10–100 µm) gap that exists between a typical shadow mask and the substrate [3]. Furthermore, flexible materials are less prone to fracture or damage during fabrication compared to rigid masks. However, lack of stiffness also implies difficulty in maintaining in-plane dimensional stability, unless a suitable (again, laborious) frame is attached. With materials as soft as PDMS (Young’s modulus ~MPa [20]; or too-thin plastic films), certain shadow mask geometries such as long suspended beam-like or cantilever-like features will additionally have insufficient out-of-plane stability. Some soft plastics like PDMS are moreover likely to contaminate the substrate, e.g., with low-molecular-weight silicone. Shadow masks from stiffer or thicker plastic films have also been used, including in combination with CO_2_ laser machining [14,21]. As seen in those examples, however, the thermal cutting process causes (1) material melting at the cut edges, forming edge beads/burr, as well as potentially inducing new residual stress in the material and (2) ejection of liquified material that can redeposit as debris on the surface—both processes ultimately degrading mask quality.

**Figure 1:**
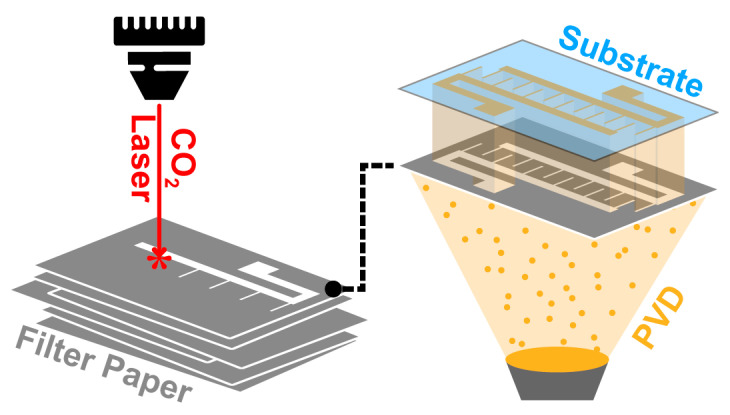
Schematic illustration of our approach. We employ a CO_2_ laser to pattern quantitative filter paper (**left**). The laser-cut paper, placed between a PVD materials source and a substrate, functions as a shadow mask for pattern transfer (**right**).

Herein, we now aim to characterize laser-cut paper as a versatile and advantageous alternative to existing shadow masks (Figure 1). Specifically, we propose that quantitative filter paper in combination with a CO_2_ laser cutter—an established approach to paper MEMS and paper microfluidics [22,23]—offers excellent synergy for SML, overcoming many of the limitations of other material/equipment combinations considered above. First and foremost, both the necessary equipment (see above) and materials (<€0.5 per 4” disc) are highly affordable and widely available. Second, quantitative filter paper, by design and definition, burns with minimal residues; we hypothesize that this makes it an ideal material for use with thermal CO_2_ laser cutting (circumventing the aforementioned limitations with plastic liquefication). Third, paper has favorable mechanical properties that make it user-friendly and resilient in handling. It is not brittle like thin crystalline membranes, eliminating the risk of shattering. Its stiffness (~GPa) is in line with (or even exceeding) that of plastic or metal foils [20,24,25], ensuring dimensional stability. Its thermal budget (~200 °C in vacuum) is higher than for many plastics [20,26]. Last but not least, the overall fabrication process is truly single-step (no tension frame mounting needed) and does not require specialized training due to most CO_2_ laser’s plug-and-play interfaces. Masks can be turned from drawings into reality in a matter of minutes. This contrasts with extensive training, experience, and processing time needed for photolithographic approaches. More advanced laser systems (e.g., “cold-cutting” ones) can have long warm-up times and tight focusing requirements, making the process more time consuming, not to mention the often-poor software associated with typical custom-built set-ups for such lasers in academic laboratories.

We note that the use of laser-cut paper masks is not inherently novel. One very recent publication using PVD to create an electrochemical oxygen sensor (>500 µm minimum feature size) references SML with laser-cut cleanroom paper in their Methods section [27]. Laser-cut paper stencils have also been used for screen printing applications (where material requirements differ somewhat from PVD) at least since 2013, showing down to 250 µm line width [28,29]. Only one of the three examples, however, fully specifies the type of paper used (revealing it as a paper/plastic hybrid, likely suffering from some of the limitations of plastics mentioned above). Critically, none of them expand on the rationale for the material choice or provide characterization results of process quality—the paper mask as an incidental process note rather than the central object of study.

Here, our focus is the thorough characterization of paper shadow mask fabrication for exemplary interdigitated electrodes (IDE) and their use in PVD processes. Ultimately, we showcase this as an ideal approach for the rapid fabrication of shadow masks towards the wide range of applications where 100 µm resolution is sufficient.

## 2. Materials and Methods

### 2.1. Design

**Figure 2:**
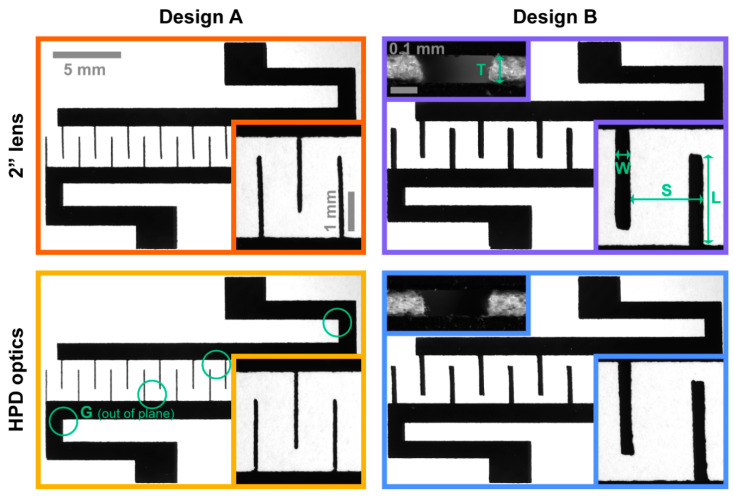
Microscopy images of exemplary laser-cut shadow masks in Whatman Grade 50 quantitative filter paper of both Designs (A: **left**; B: **right**), utilizing either of the available lenses (2”: top; HPD: bottom). The square insets show electrode finger details (top right further indicating the analyzed dimensions; cf. Table 2). For Design B, we additionally show insets of razor-blade-cut mask cross-sections of a single electrode finger, sandwiched between two glass slides, indicating the mask thickness *T* (cf. Table 3). Scale bars are conserved across all four conditions. The green circles (bottom left) show selected measurement locations for the out-of-plane gap *G* between the mask and the underlying substrate (Figure 3 and Section 3.3). The border colors correspond to those used for the data in Figure 4.

We considered two IDE mask Designs, A and B, that allowed us to assess a range of quality parameters for wafer-scale masking that translate to generic features. Nominal IDE dimensions are summarized in Table 2 (and indicated in Figure 2) with the elemental IDE design unit (footprint ~12 × 20 mm) repeated 12 times across a single mask (typical 4-inch wafer area).

### 2.2. Materials

We evaluated quantitative cellulose filter papers from Whatman (GE Life Sciences; now Danaher Corp., Washington, DC, USA), Grades 50 and 540, and Sartorius (Göttingen, Germany), Grades 392 and 393. Their selection is further discussed in Section 3.1. We used 25 µm polycarbonate membranes (it4ip; Louvain-la-Neuve, Belgium) as substrates for subsequent deposition.

### 2.3. Laser Cutting

We employed a VLS 2.30 CO_2_ laser cutter/engraver (10.6 µm wavelength; Universal Laser Systems, Scottsdale, AZ, USA) for our study. A relatively high-end instrument, it features both a high-power density lens (HPD; nominal 25 µm spot size) as well as a 2” focal length lens (nominal 125 µm spot size) that is also common in more affordable instruments. Cutting power, speed, and focal plane were iteratively optimized to achieve the thinnest possible continuous cut lines. The optimal parameters all utilized a solid cutting support (e.g., Si wafer; cutting on honeycomb support resulted in defects where the beam crossed the lattice), with lateral air assist, 900 mW (HPD: 960 mW) laser power at 1000 pulses/in and 0.45 in/s (0.4 in/s) linear movement speed, and the highest vector quality setting available in the software.

### 2.4. PVD

For metal deposition, we employed electron beam evaporation in a PAK 600 Coating System (Provac GmbH; Sprendlingen, Germany). Besides general considerations of chamber geometry for SML (cf. Section 3.3), the main PVD requirements are chamber pressure and temperature in line with material limitations. In our electron beam-based process, the sample temperature was maintained near room temperature (<50 °C) at ~0.6 mPa, but other processes (thermal evaporation, pulsed laser deposition, and so on.) could vary widely in these parameters. Paper masks remain unaffected up to ~200 °C in vacuum [26], allowing also for higher-temperature processes than ours. Many organics that might be utilized as either substrate or an alternative masking material (e.g., polycarbonate) have lower thermal budgets than this [20], with biological materials likely faring even more poorly. In such cases, which constitute perhaps the most interesting areas of application, the paper mask itself will not be the limiting factor.

The polycarbonate membrane substrate was cut to the desired size (using the same CO_2_ laser cutter) and ultrasonicated in isopropanol for 1 min, followed by immersion in deionized water and gentle drying with an air gun. We then mounted the substrate underneath the laser-cut paper mask in a custom computer numerical control (CNC)-machined aluminum rig and fixed this above the PVD materials source in the deposition chamber. Typical deposition parameters were 15 nm titanium adhesion layer (0.5 Å/s) and 170 nm gold (2 Å/s), without sample rotation.

### 2.5. Lateral Characterization

For lateral structural evaluation, we relied on a Leica variable-magnification stereomicroscope. We selected *n* = 12 IDEs per Design and lens type at random (across eight independently fabricated masks), acquiring both low- and high-magnification images. We analyzed images in Fiji [30], with our process flow illustrated and expanded on in the supplementary materials. In brief, images were manually corrected for rotation using TransformJ [31] and thresholded using the Li algorithm [32]. We subsequently selected the relevant areas of interest and extracted Plot Profiles, which we further processed in MATLAB (MathWorks, Natick, MA, USA) to extract width (direct mathematical conversion; Figure S1) as well as length and spacing parameters (based on edge detection; Figure S2). Imaging of a calibration scale allowed us to convert pixel distances into absolute units.

### 2.6. Vertical Characterization

Filter paper thickness was measured with a plunger-type mechanical dial indicator at *n* ≥ 5 independent samples across filter discs per paper type.

To characterize filter paper surface morphology, we relied on a P15 surface profiler (KLA/Tencor, Milpitas, CA, USA), executing *n* = 3 line scans of 5 mm each per paper type at different spots on the filter discs. A software filter with ~1 mm wavelength was applied to the raw data to extract root mean square (RMS) waviness (i.e., variability of the filtered data, indicating millimeter scale texture) and RMS roughness (i.e., variability of the filter-subtracted data, indicating microscale texture) [33].

To assess conformality, we mounted shadow masks of both Designs into our deposition rigs and measured vertical profiles at four spots per IDE (cf. Figure 2; total *N* = 94) with a WYKO NT9300 optical profilometer (Veeco, Plainview, NY, USA). Spots were selected to capture maximum expected mask deflection. Sample readings and processing are detailed in Figure S3.

### 2.7. Statistics and Data Availability

All statistical analyses were conducted using Origin Pro (Originlab, Northampton, MA, USA). Raw data were deposited in a freely available repository [34].

## 3. Results and Discussion

### 3.1. Materials Selection

The ideal shadow mask is infinitely thin and in infinitely close contact with the substrate. Intuitively, this can be understood in similar terms to photolithography, where a mask–substrate gap leads to diffraction artifacts. Mathematically, how well a feature or electrode *E* is transferred by PVD from the shadow mask onto the substrate (as a “blurred” *E’*) is described by [35]:

*E*’ = [*G* ⋅ (*Ø* + *E*) + *D* ⋅ *E* – *Ø* ⋅ *T*/2]/[*D* + *T*/2] (1)

Relevant variables are (1) the size *Ø* and distance *D* of the (directional) deposition source, discussed more in Section 3.3; (2) the thickness *T* of the mask; and (3) the gap *G* separating it from the substrate.

**Figure 3:**
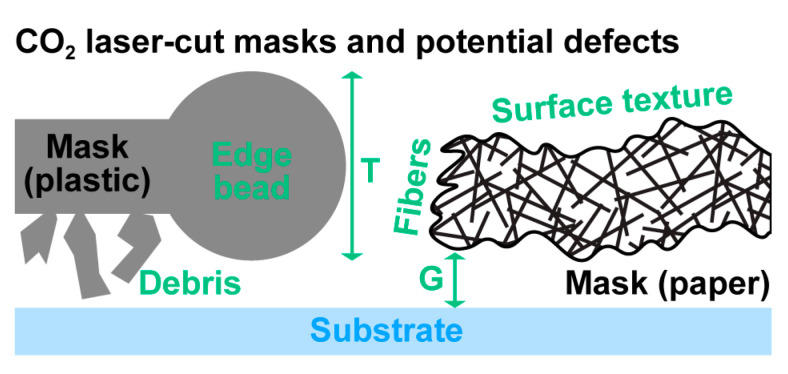
Schematic illustration of shadow masks and how various defects can impact patterning quality by increasing mask thickness and/or the gap between mask and substrate.

As laid out in the Introduction, CO_2_ lasers are both affordable and suitable for cutting a wide range of materials. This includes many plastics that are available cheaply as thin films. However, plastic does not vaporize cleanly; at the submillimeter scales we are aiming for, we find that even acrylic, generally regarded as a superior laser cutting plastic [36], suffers from this limitation. The resulting edge deformation and particulate debris both adversely affect mask thickness *T* and gap *G* (Figure 3).

We hypothesize that quantitative filter papers make for near-ideal CO_2_ laser cutting materials due to their (by definition) exceptionally clean burn [37,38], eliminating the plastic-associated limitations. Yet paper is a complex heterogeneous material that could present a different set of artifacts. If the fibers are too sparse within the paper matrix, cut edges could be very rough (both vertically as shown in Figure 3, as well as laterally) as air pockets alternate with cut fibers. Additionally, paper can have a pronounced surface texture, which could adversely affect gap distance.

For our study, we selected four candidate quantitative filter papers from two manufacturers (Table 3). They were selected toward the low range of available thicknesses within the relevant catalogs [37,38], as well as for high density. Low thickness is clearly advantageous from Equation (1), while high volumetric density implies a denser fiber network with fewer air pockets that would increase cut roughness. Surface smoothness is not directly specified by the manufacturer; hence, we subjected all samples to profilometry.

The hardened Whatman Grade 50 proved superior to the other candidates across all measures we considered. This implies more homogenous cutting properties as well as better contact with an underlying substrate in shadow mask processes. Whatman Grade 50, moreover, features minimal fiber shedding and hence, recommended for cleanroom processing by the manufacturer, eliminating at least some of the concerns where the introduction of paper into clean environments and processing tools was concerned [37]. Last but not least, being a dense and hardened filter paper, Whatman Grade 50 likely has a Young’s modulus in excess of the ~1.5 GPa reported for standard quantitative filter paper [24]. This was supported by qualitative beam bending observations comparing cantilevers cut from Whatman Grade 50 with 125 µm thick polycarbonate (~2.3 GPa; typical for many plastics [20]) film. The filter paper consistently deflected less under its own weight as well as under external load, both in vertical and lateral directions. The paper ultimately showed high-quality laser cuts without visible material deformation (Figure 2 cross-sections) or particulate redeposition and was used for all further analysis.

### 3.2. Cutting Quality

When considering the quality of our laser-cut shadow masks, we can separate two distinct components. First, the edge and line quality (their smoothness or roughness); we can expect this to be governed mainly by the laser spot quality, i.e., the lens utilized. Second, the positional accuracy of features with relation to one another; this is likely to be more a function of the motorized stage. Our IDE features and designs give insight into these distinct components.

A general idea of quality can be gained from images like those shown in Figure 2, especially when considering Design A with its IDE fingers consisting of single lines. We observed that the 2” lens yields rougher-looking cuts, whereas the HPD lens produced smoother and somewhat thinner ones, broadly in line with expectations. However, more in-depth quantitative analysis is clearly required. For this, we focused on assessing absolute Deviation from the nominal parameters listed in Table 2. Deviation is independent of Design variations and allows for comparison across the different parameters that we employed to analyze quality and accuracy.

Finger width *W*—line quality and submillimeter accuracy

**Figure 4:**
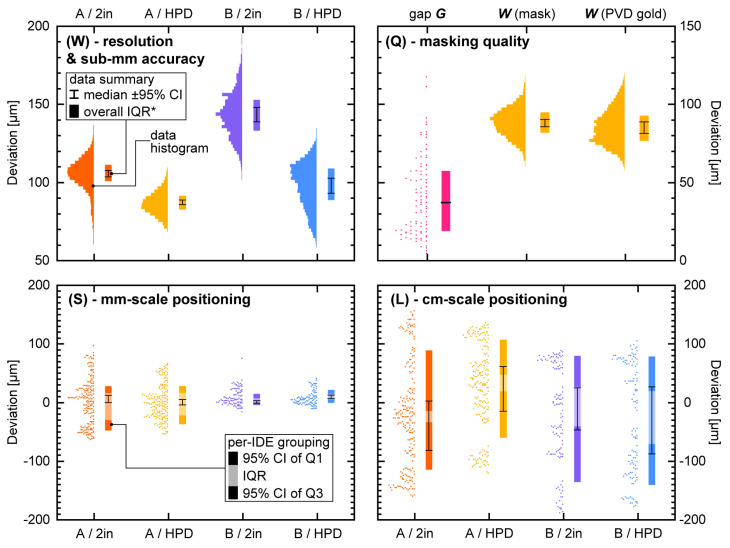
Deviation from nominal design targets (cf. Table 2) for a range of parameters, for (**W**,**S**,**Q**) as a function of Design and lens type. Colors (except magenta) correspond to those in Figure 2. The graphs show data distributions (histograms if *n* > 10,000) and corresponding summary parameters (median; interquartile range IQR; where applicable also 95% confidence intervals (CIs) and first/third quartiles (Q1/Q3)). To derive CIs, data are grouped either on per-finger (**W**,**Q**; *n*(A) = 48; *n*(B) = 24) or per-IDE (**S**,**L**; *n* = 12 each) basis. (**W**) IDE finger width (at each pixel along finger length; *n*(A)~48 × 830; *n*(B)~24 × 1060), in Design A corresponding to the laser line width. IQRs* are for overall distribution (cf. main text). (**S**) IDE finger spacing (for each finger; *n*(A)~15.5 × 12; *n*(B)~8 × 12). (**L**) IDE finger length (for each finger; *n*(A)~17 × 12; *n*(B)~10 × 12). (**Q**) (left) Gap *G* between mask and substrate (at four points per IDE; *N* = 94; no grouping). (right) IDE finger width for a Design A mask written with HPD optics as well as corresponding PVD electrodes (*n*~24 × 760).

In Figure 4W, we display histograms of electrode finger widths *W* obtained with either lens, along with summary parameters—medians, interquartile ranges (IQRs), and relevant 95% confidence intervals (CIs) where applicable. As described in Section 2.5 and Figure S1, the higher-resolution images of the fingers (Figure 2 insets) allowed us to extract width at each pixel along their length. The width is given in terms of Deviation from the nominal size (cf. Table 2)—in Design A with 0 µm nominal width, therefore, capturing the intrinsic line quality. The 2” lens produced lines of 105 µm median width (95% CI: 103 to 107), even slightly below the nominal 125 µm spot size. Our result is also superior to previous work with Whatman Grade 50 for microfluidic applications [23]. The HPD lens yielded significantly thinner lines of only 87 µm (95% CI: 86 to 89). Interestingly, this is worse than the nominal 25 µm spot size, indicating that the limiting factor here is not the lens. Instead, it appears that thermal transport in the paper led to burning/vaporization of a roughly 3× wider area.

Besides the line thickness, we can also determine line roughness, i.e., how much the line width varies within each individual finger. The 2” lens provided a per-finger interquartile range (IQR; not displayed on graph) of 9.8 µm (95% CI: 8.9 to 10.7), whereas the HPD optics produced 7.2 µm (95% CI: 6.7 to 7.6). This 27% improvement aligns with the qualitative observations as well as expectations and is somewhat more pronounced than the 17% improvement in line width. Overall, however, using a cheap and widely accessible 2” lens laser cutter appears to still provide acceptable quality.

Figure 4W also includes the relevant data for Design B. Here, fingers were written as a box with nominal width of 200 µm; a 100 µm Deviation value thus corresponds to a 300 µm measured electrode finger width. The Deviation here is thus a combined function of line quality (as for Design A), plus submillimeter positioning accuracy (the two sides of the “box”). We would expect accuracy to mostly affect the spread of the distributions, resulting in sometimes wider and sometimes thinner IDE fingers. Indeed, the histograms show a roughly two-fold increase in the overall (rather than per-finger) IQR* from 9 to 10 µm (Design A) to 20 µm. The 10 µm difference yields a measure for submillimeter positioning accuracy.

The more visually obvious difference of the histograms, however, is an increase in median Deviation (95% CIs are nonoverlapping for all conditions), particularly with the 2” lens. Although a constant positive positioning offset is conceivable, if present it should be conserved for both lenses. Instead, we theorize that the second (parallel) pass in Design B burned away additional material also at the exposed edge from the first line. The more confined beam of the HPD lens (with single line width as mentioned before reliant on thermal transport inside the paper matrix) would, however, have a more limited effect across the air gap between lines compared to the 2” lens, matching the observations. Narrowly (200–400 µm) spaced lines will thus benefit from an HPD lens, whereas wider spacings can be achieved with either.

Finger spacing *S*—millimeter-scale accuracy

The spacing *S* of our IDE fingers in both Designs can give us an idea of positioning accuracy on larger length scales than (and without the confounding factors of) finger width *W*. We show the relevant data—again as the Deviation from nominal (cf. Table 2)—in Figure 4S in terms of both actual distributions as well as summary parameters. The analysis process, based on the lower-magnification images (Figure 2), is illustrated in Section 2.5 and Figure S2. Median Deviations, here, are close to zero in all conditions (<8 µm, below the resolution limit of the *S* and *L* analysis). This aligns with our expectations for positional accuracy quantification as discussed above. The IQR (grouped per IDE in order to derive CIs) shows no significant differences based on lens type (substantial overlap in 95% CIs), but interestingly decreases based on Design from 56 (A; 95% CI: 51 to 62) to 15 µm (B; 95% CI: 12 to 17). The latter is very close to the 10 µm positional accuracy seen above for 200 µm distances, in spite of the roughly 10 times larger distances involved. We speculate that the larger IQR for Design A is partly due to the analytical algorithm being less accurate in detecting the two closely (<10 pixel) spaced edges of thin IDE fingers. The different laser head movement paths in writing single lines versus box electrodes may also contribute. The overall average IQR* of 35 µm (95% CI: 27 to 42) likely presents a reasonable estimate for most scenarios of millimeter-scale accuracy.

Finger length *L*—centimeter-scale accuracy

We display data on the IDE finger length *L* in Figure 4L, wholly analogous to Figure 4S. While the nominal lengths are millimeter-scale (cf. Table 2), we can use the Deviation for this case to infer wafer-scale positioning accuracy. This is because the dominant factor for length is the placement of the large bars/contact pad traces at the top and bottom, which for the purposes of this study, we chose to write after IDE fingers have been patterned over the entire ~4”-wafer-size mask area, i.e., after the print head has traversed a cumulative >50 cm. This is supported by per-IDE histograms showing bimodal characteristics. In the overall distributions of Figure 4L, this is still apparent from the distinct pillars and gaps, as well as the large uncertainty in IQR (grouped per IDE). Although the median values vary between conditions, their confidence intervals include zero and largely overlap (as do those of the IQRs). Grouping all data together, we establish an IQR* of 116 µm (95% CI: 96 to 135). This is substantially larger than the millimeter-scale values above. However, considering the over 100-fold larger distances involved, this worst-case positional accuracy should still be acceptable for many applications such as discussed in the Introduction.

### 3.3. Masking Quality

The quality of the shadow mask is critical to ensure good patterning of the desired features. Pattern transfer onto the substrate also depends on the parameters in Equation (1), from which, we can derive some general implications. First, a deposition method with large *D* and small *Ø* is desirable, such as our electron beam evaporation chamber with *D*~0.5 m and *Ø*~1 cm (sputter deposition, which employs short *D* and large *Ø*, is not advisable). Second, a thicker mask will cause shadowing (decrease in *E*’), which informed our choice of material as discussed in Section 3.1. Due to the laser creating a V-shaped sidewall profile (Figure 2 cross-sections), the effective *T* (for the purposes of deposition quality) may be even lower than those measured in Table 3. Third, the substrate–mask distance will increase blurring, for our deposition chamber geometry by roughly 2% of *G*. We note that a second mechanism, the halo effect, also impacts blurring—empirically, this depends largely on specific PVD parameters, as well as increasing with *G* [35]. Haloing is not expected to impact an optical analysis such as ours, however.

In Figure 4Q, we display measurements of *G* with our laser-cut shadow masks mounted in the custom-made deposition rig, which keeps substrate and mask in contact. Our rig was CNC machined, though 3D printing can likely provide an equally suitable alternative. The measurement locations were chosen at spots where we may expect the largest gaps, i.e., where surrounding paper provides the least structural support—the ends of long cantilever-like mask structures and the center of long beam-like mask structures (indicated by circles in Figure 2). The analysis process is described in Section 2.6 and Figure S3. We thereby obtained a median measured gap of 37 µm, with an overall IQR* of 38 µm. We note that the gap in more “constrained” spots of the design tends toward <10 µm, i.e., more closely matching the expected gap from the surface roughness measurements (Table 3). Our quantified gaps instead are dominated by the dimensional stability of the masks. Although the IQR of traditional shadow masks is likely smaller, our values are well within the typical range of *G*~10–100 µm [3].

Finally, we subjected a set of Design A IDEs (HPD lens) patterned in PVD gold onto a polycarbonate membrane to the same finger width analysis as their corresponding masks, displayed side-by-side also in Figure 4Q. We note that these masks were written with somewhat less optimized parameters than those presented in Section 3.2. The confidence intervals of the medians largely overlap, which is consistent with Equation (1) predicting a shift for the median in the range of −0.5 to +1 µm (due to the uncertainty in effective *T* mentioned above). Notably, the distribution for the gold IDEs is wider, with overall IQR increasing by 25% compared to the mask, from 13.0 to 16.1 µm. This is consistent with the variability inherent in the gap, combined with the more diffuse edge definition from blurring making our thresholding analysis less reliable.

## 4. Conclusions

We have presented and characterized a highly affordable method for production of PVD shadow masks. We found quantitative filter papers, particularly hardened low-ash Whatman Grade 50, to be well-suited to shadow mask fabrication via CO_2_ laser cutting. The process is fast (<10 min per 4” mask) and affordable both in material (<€0.5 per mask) and equipment (from €100 used). This is in stark contrast to traditional solid-state masks that are very costly to purchase and time consuming to produce. Compared to many plastic films (which could be processed similarly), paper eliminates laser cutting artifacts that can degrade mask quality and features superior mechanical properties. We characterized, for the first time, the resolution and accuracy of our process and find that roughly 100 µm line widths can be achieved with both standard 2” and high-end HPD optics. Accuracy of the process increases with length scale, ranging from 10 (submillimeter) to 120 µm (wafer-scale). Although this is significantly worse than the micron-size features of traditional stencils, the submillimeter performance we demonstrated is sufficient for a wide range of applications. We further confirmed that our masks can be put into close (<100 µm) contact with substrates and that PVD feature transfer from mask to substrate matches expectations for a shadow mask process. In conclusion, we believe this method can significantly reduce costs and speed up design cycles for a wide range of applications where submillimeter PVD features on organic or biological substrates are desired—from three-electrode electrochemical cells to contact pads for organic electronic materials.

## Figures and Tables

**Table 1:** Overview of select shadow mask fabrication approaches.

	Photolithography	“Cold” Laser Machining	CO_2_ Laser Cutting	
Tools	Full cleanroom	>€10,000 laser	>€100 laser	
Typical materials	Si, SiN, metal	Metal, plastics	Plastics	**Filter paper (our work)**
Thickness	<1 µm	<10 µm	~100 µm	~100 µm
Process cost ^$^	>€1000	>€10–100	<€1	<€1
Process complexity	Complex	Complex mounting	Simple, single-step	
Process artifacts	None	None	Particle ejecta, edge deformation	None
Mask handling	Fragile (brittle)	Fragile (thin)	Potential x/y/z distortion *	Resilient
Resolution	<1 µm	<10 µm	~100 µm	87 µm line width, ±58 µm accuracy (wafer-scale)

^$^ Process cost is directly proportional to process time. * Mask distortion for plastic films depends on their thickness and inherent stiffness.

**Table 2:** Summary of nominal design parameters for the critical interdigitated electrodes (IDE) finger dimensions.

Design	Width *W*	Length *L*	Spacing *S*
A	0 µm *	1875 µm	1000 µm
B	200 µm	2375 µm	1800 µm

* A nominal width of zero, in practice, will result in the minimum possible line width achievable with the laser.

**Table 3:** Summary of quantitative filter properties considered. The most favorable values (highest for density; lowest for all others) are bolded in each column.

Type	Density ^1^	Nominal *T* ^1^	*T* ^2^	Roughness ^2^	Waviness ^2^
**Whatman 50**	**0.83 g/cm³**	**115 µm**	**98 ± 5 µm**	**3.6 ± 0.4 µm**	**1.1 ± 0.2 µm**
Whatman 540	0.53 g/cm³	160 µm	128 ± 8 µm	7.0 ± 1.5 µm	4.1 ± 2.9 µm
Sartorius 393	0.59 g/cm³	170 µm	135 ± 3 µm	4.5 ± 0.5 µm	2.1 ± 0.3 µm
Sartorius 392	0.49 g/cm³	170 µm	126 ± 3 µm	11.4 ± 1.7 µm	7.2 ± 2.2 µm

^1^ From manufacturer data sheets [37,38]. ^2^ Measured data (cf. Section 2.6). For thickness (*n* ≥ 5), local compression of the paper by the measurement probe may explain the discrepancy compared to nominal. Roughness and waviness are RMS values (*n* = 3 × 5 mm) for surface texture smaller and larger than ~1 mm, respectively.

## Supplementary Materials

The following are available online at https://www.mdpi.com/2072-666X/11/7/676/s1, Figure S1: Finger Width Analysis, Figure S2. Spacing and Length Analysis, Figure S3. Gap Analysis.
