# Supplementary material for: Low-Cost PVD Shadow Masks with Submillimeter Resolution from Laser-Cut Paper
Source: Micromachines (Basel). 2020 Jul 11;11(7):676. doi: 10.3390/mi11070676 (PMC7407583; doi:10.3390/mi11070676)
Supplement: Supplementary file 1 [file micromachines-11-00676-s001.pdf]

micromachines

Supplementary Materials

Low-cost PVD shadow masks with sub-mm resolution from laser-cut paper

Farzad Elhami Nik,[1,2] Isabelle Matthiesen,[1] and Anna Herland[1,3*] Thomas E. Winkler[1*]

1 Division of Micro- and Nanosystems, KTH Royal Institute of Technology, Stockholm, Sweden.
2 Department of Electronics, Information and Bioengineering, Politecnico di Milano, Milan, Italy.
3 AIMES, Department of Neuroscience, Karolinska Institute, Solna, Sweden.
* Correspondence: winklert@kth.se; aherland@kth.se

Figure S1. Finger Width Analysis

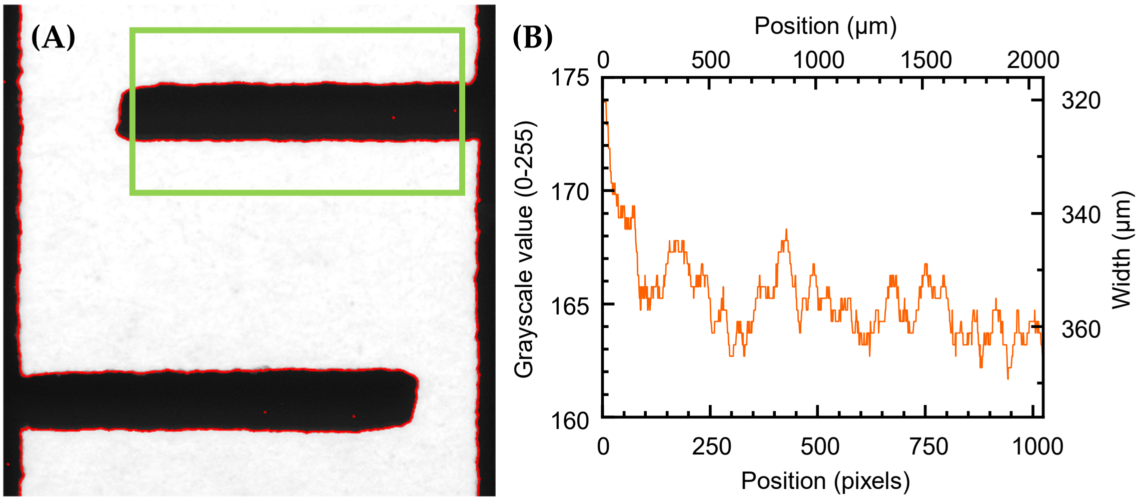

A: High-magnification micrograph of two electrode fingers (same as Figure 2, B / 2in), straightened using TransformJ [31]. The auto-thresholding by the Li algorithm [32] (which, in the analysis workflow, converts the electrode fingers to solid black, the mask itself to solid white) is indicated with the red edge outline.

B: Fiji Plot Profile from the green-outlined selection area, taken along horizontal axis. The orange line captures the vertical-averaged grayscale value of the (thresholded) image at each pixel position, *i.e.* n~800–1000 per finger. Since the selection height is known, and the black (zero-value) pixels correspond to the electrode finger, grayscale values (left axis) can be converted to width W (right axis) using:

$$W = (1 - (\text{grayscale value} / 255)) \times \text{selection height [µm]}$$

Subtracting nominal width (Table 2) from the measured parameter yields the Deviation. Thus we obtain a histogram of W for each finger (to derive per-finger IQR) and can combine all measured fingers for each condition into the overall histograms in Figure 4.

Figure S2. Spacing and Length Analysis

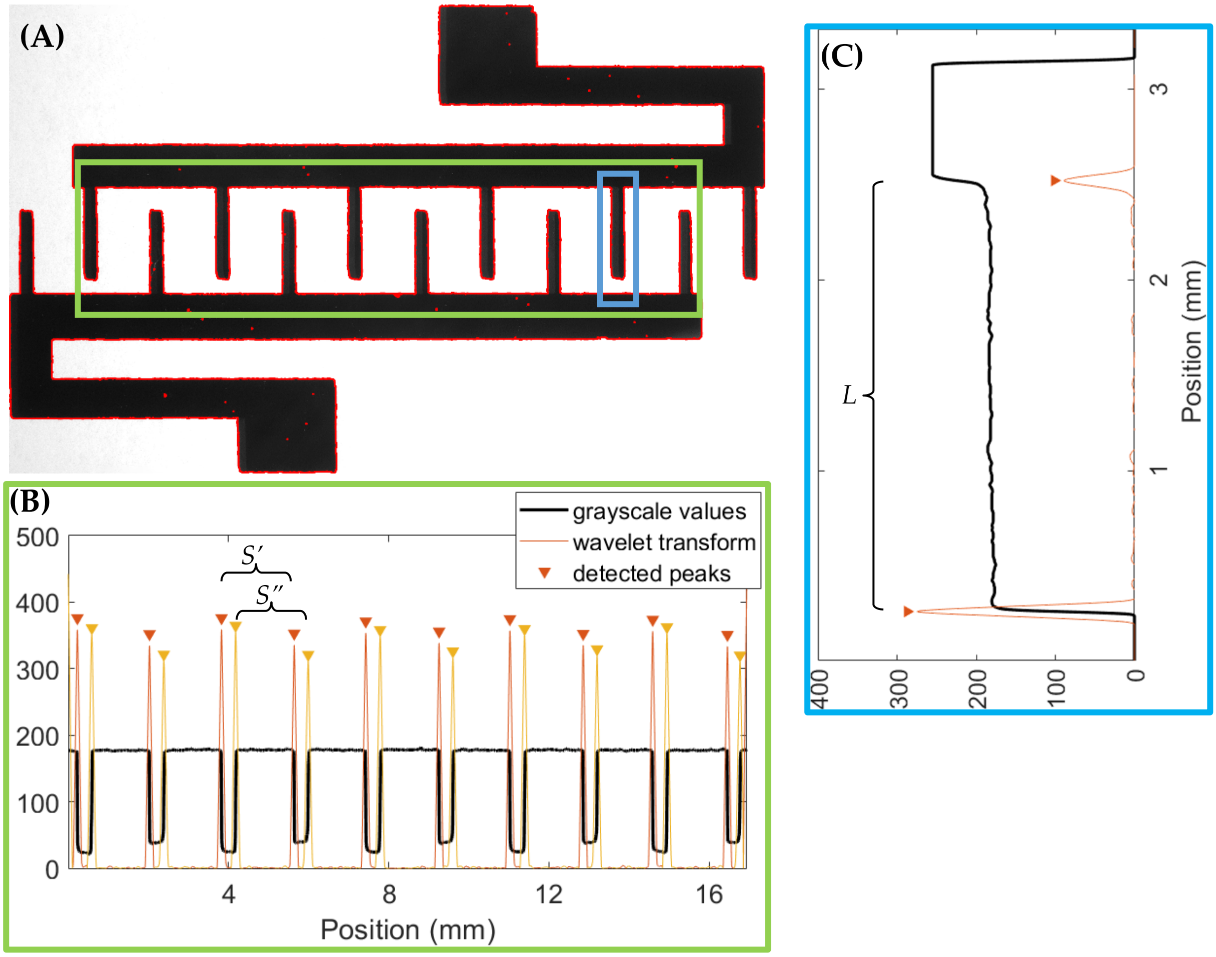

A: Low-magnification micrograph of IDE mask (same as Figure 2, B / 2in), straightened using TransformJ [31]. The auto-thresholding by the Li algorithm [32] (which, in the analysis workflow, converts the electrode fingers to solid black, the mask itself to solid white) is indicated with the red edge outline.

B: Fiji Plot Profile from the green-outlined selection area, taken along horizontal axis, with arbitrary y-axis. The black line captures the vertical-averaged grayscale value of the (thresholded) image at each horizontal pixel position. The red and yellow line show the MATLAB-determined continuous Haar wavelet transform (wavelet scale: ~100 µm) of the black line, with peaks (corresponding to falling and rising edges) marked by triangles. IDE spacing S is calculated for each electrode pair as:

$$S = (S' + S'') / 2$$

Subtracting nominal spacing (Table 2) from the measured parameter yields the Deviation. We thus obtain n~8–16 values per IDE (used in some statistical analysis), which we further combine into the overall per-condition histograms shown in Figure 4.

C: Fiji Plot Profile from the blue-outlined selection area, taken along vertical axis, with arbitrary x-axis. Grayscale values and wavelet transform shown and analyzed as in sub-figure B. Electrode length L is calculated as the distance between peaks as indicated. Subtracting nominal length (Table 2) from the measured parameter yields the Deviation. We repeat this for each finger per IDE, yielding grouped and ultimately overall distributions.

Figure S3. Gap Analysis

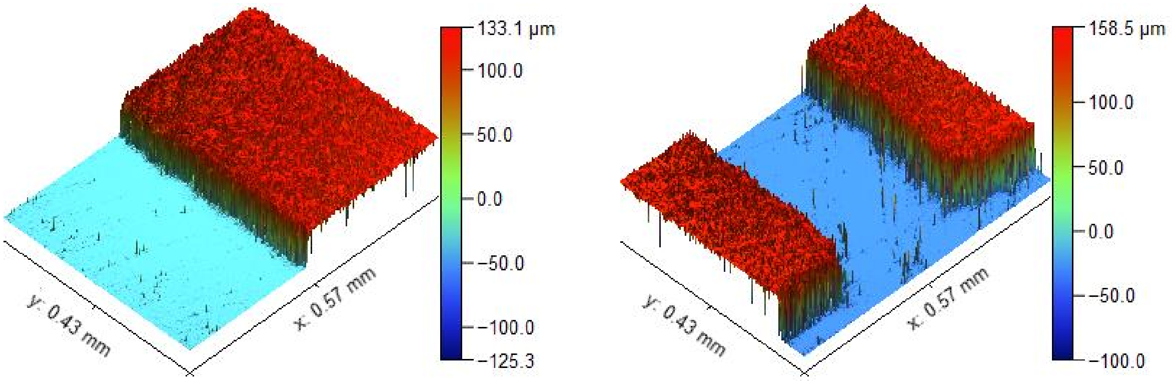

Exemplary optical profilometry results (left: "cantilever" endpoint; right: "fingers"; positions indicated in Figure 2 with green circles). The substrate shows up in blue, the top of the mask in red. To extract gap G, we first convert the displayed height data into histograms, which will show two distinct peaks (*i.e.*, substrate plane and top of mask). The average vertical positions V' and V'' of the planes are extracted by local weighted linear smoothing (~25 µm window) and peak finding in MATLAB. Accounting further for the known thickness T of the mask itself (Table 2), we thus obtain:

$$G = V'' - V' - T$$

© 2020 by the authors. Submitted for possible open access publication under the terms and conditions of the Creative Commons Attribution (CC BY) license (http://creativecommons.org/licenses/by/4.0/).
